# Supplementary material for: Enhancement of Photodetector Characteristics by Zn-Porphyrin-Passivated MAPbBr3 Single Crystals
Source: Nanomaterials (Basel). 2024 Jun 21;14(13):1068. doi: 10.3390/nano14131068 (PMC11243306; doi:10.3390/nano14131068)
Supplement: Supplementary file 1 [file nanomaterials-14-01068-s001.zip › nanomaterials-3055291-supplementary.pdf]

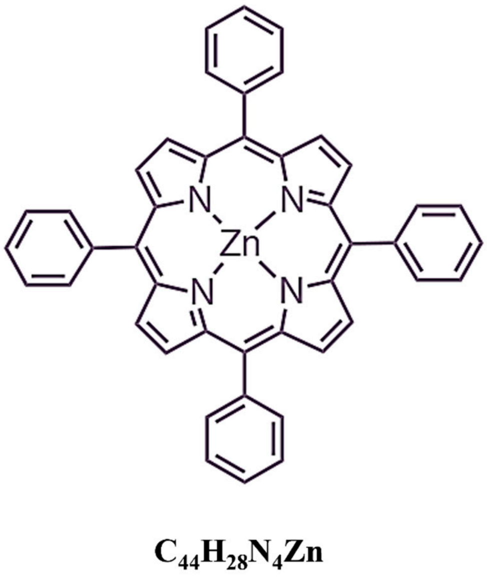


**Figure S1.** Chemical structure and formula of Zn-PP.

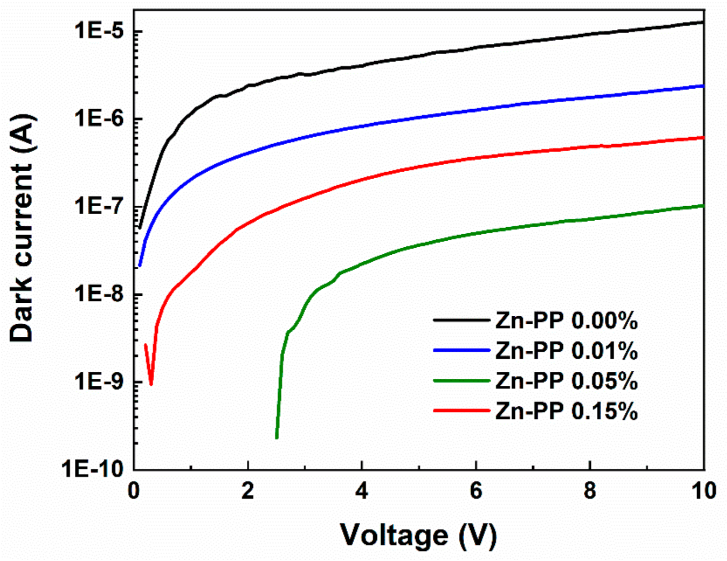


**Figure S2:** I-V curve of $MAPbBr_3$ SC photodetectors performed under dark conditions.

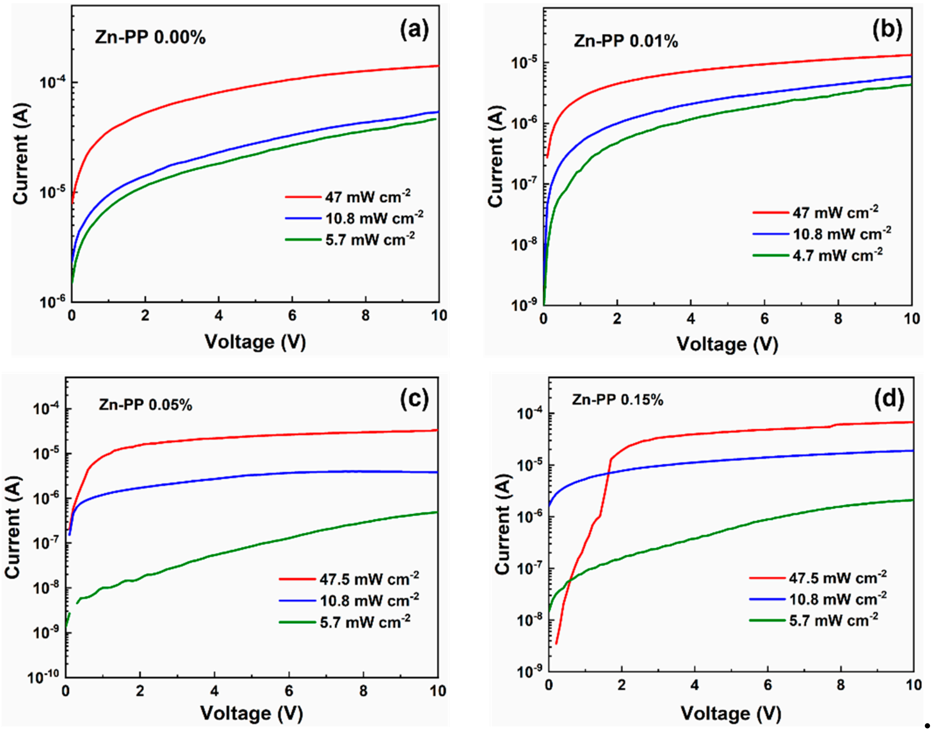


**Figure S3:** The current-voltage curves of $MAPbBr_3$ single crystals measured under different light intensities.

**Table S1.** The comparison of the performance parameters for $MAPbBr_3$ SC and metal-doped $MAPbBr_3$ SC-based PDs reported in the literature and our work.

| Device Configuration | Applied bias &light source | Responsivity ($AW^{-1}$) | Detectivity (Jones) | EQE (%) | Reference |
|---|---|---|---|---|---|
| $MAPbCl_3$-$MAPbBr_3$/Cu | 5V & 400 nm ,1.64 mW/cm$^2$ | 0.614 | 1.03 x $10^{12}$ | — | [1] |
| Pt/$CH_3NH_3PbBr_3$/Au | 3V & white light, 100 mW/cm$^2$ | 0.1 | 7.1 x $10^{11}$ | — | [2] |
| PET/$MAPbI_3$/PMAA/Au | 10V & 532 nm, 100μW/cm$^2$ | 0.1 | 1.02 x $10^{12}$ | — | [3] |
| Pt / $MAPbBr_3$ / Pt | 2V & 530 nm, 0.1 mW/cm$^2$ | 3.31 | 7.02 x $10^{12}$ | 773 | [4] |
| Au / Er:$MAPbBr_3$ / Au | 4V & 520 nm, 20 μW cm$^{-2}$ | 10 | 2.02 x $10^{12}$ | 2500 | [5] |
| Zn-PP:$MAPbBr_3$/Au | 5V, 405 nm, 47 mW/cm$^2$ | 5.16 | 4.76 x $10^{12}$ | 1581 | This work |

## References


1. Qiu, X.; Wang, Y.; Li, M.; Huang, L.; Yang, J.; Li, G.; Zhang, X.; Xiao, K.; Sun, W. Improved optoelectronic performance from the internal secondary excitation of MAPbCl3-MAPbBr3 single crystal photodetectors. *Ceramics International* **2023**, *49*, 518-527, doi:https://doi.org/10.1016/j.ceramint.2022.09.019.
2. Chen, L.; Wang, H.; Zhang, W.; Li, F.; Wang, Z.; Wang, X.; Shao, Y.; Shao, J. Surface Passivation of MAPbBr3 Perovskite Single Crystals to Suppress Ion Migration and Enhance Photoelectronic Performance. *ACS Applied Materials & Interfaces* **2022**, *14*, 10917-10926, doi:10.1021/acsami.1c21948.
3. Deng, H.; Yang, X.; Dong, D.; Li, B.; Yang, D.; Yuan, S.; Qiao, K.; Cheng, Y.-B.; Tang, J.; Song, H. Flexible and Semitransparent Organolead Triiodide Perovskite Network Photodetector Arrays with High Stability. *Nano Letters* **2015**, *15*, 7963-7969, doi:10.1021/acs.nanolett.5b03061.
4. Anilkumar, V.; Mahapatra, A.; Nawrocki, J.; Chavan, R.D.; Yadav, P.; Prochowicz, D. Metal-Doped MAPbBr3 Single Crystal p-n Junction Photodiode for Self-Powered Photodetection. *Advanced Optical Materials* **2024**, *12*, 2302032, doi:https://doi.org/10.1002/adom.202302032.
5. Rong, S.; Xiao, Y.; Jiang, J.; Zeng, Q.; Li, Y. Strongly Enhanced Photoluminescence and Photoconductivity in Erbium-Doped MAPbBr3 Single Crystals. *The Journal of Physical Chemistry C* **2020**, *124*, 8992-8998, doi:10.1021/acs.jpcc.0c01959.
